# Essential tremor

**DOI:** 10.1186/2050-5736-3-S1-O1

**Published:** 2015-06-30

**Authors:** Jeff Elias

**Affiliations:** 1 University of Virginia, Charlottesville, Virginia, United States

## Background/introduction

A randomized controlled trial of unilateral stereotactic MRI-guided focused ultrasound thalamotomy is underway.

## Methods

Seventy-two patients with severe, medication-refractory essential tremor have been enrolled across seven international centers. Standard assessments of tremor severity and disability will be obtained before and after the procedure. Patients are randomized in a one-to-three fashion to receive either sham procedure or MR-guided focused ultrasound thalamotomy. The Tremor Research Group, neurologist specialists in the management of essential tremor, will rate the clinical outcomes from videotaped assessments.

